# Exploratory Analysis of Fast-Food Chain Restaurant Menus Before and After Implementation of Local Calorie-Labeling Policies, 2005–2011

**DOI:** 10.5888/pcd10.120224

**Published:** 2013-06-20

**Authors:** Alexa Namba, Amy Auchincloss, Beth L. Leonberg, Margo G. Wootan

**Affiliations:** Author Affiliations: Alexa Namba, School of Public Health, Drexel University, Philadelphia, Pennsylvania; Beth L. Leonberg, College of Nursing and Health Professions, Drexel University, Philadelphia, Pennsylvania; Margo G. Wootan, Center for Science in the Public Interest, Washington, DC

## Abstract

**Introduction:**

Since 2008, several states and municipalities have implemented regulations requiring provision of nutrition information at chain restaurants to address obesity. Although early research into the effect of such labels on consumer decisions has shown mixed results, little information exists on the restaurant industry’s response to labeling. The objective of this exploratory study was to evaluate the effect of menu labeling on fast-food menu offerings over 7 years, from 2005 through 2011.

**Methods:**

Menus from 5 fast-food chains that had outlets in jurisdictions subject to menu-labeling laws (cases) were compared with menus from 4 fast-food chains operating in jurisdictions not requiring labeling (controls). A trend analysis assessed whether case restaurants improved the healthfulness of their menus relative to the control restaurants.

**Results:**

Although the overall prevalence of “healthier” food options remained low, a noteworthy increase was seen after 2008 in locations with menu-labeling laws relative to those without such laws. Healthier food options increased from 13% to 20% at case locations while remaining static at 8% at control locations (test for difference in the trend, *P* = .02). Since 2005, the average calories for an à la carte entrée remained moderately high (approximately 450 kilocalories), with less than 25% of all entrées and sides qualifying as healthier and no clear systematic differences in the trend between chain restaurants in case versus control areas (*P* ≥ .50).

**Conclusion:**

These findings suggest that menu labeling has thus far not affected the average nutritional content of fast-food menu items, but it may motivate restaurants to increase the availability of healthier options.

## Introduction

Beginning with New York City in 2008, 18 states and localities have implemented regulations requiring chain restaurants to post calorie information on menus and menu boards (1). This approach to addressing obesity aims to assist customers in making informed, lower-calorie choices. Although it is too early to assess the full impact of menu labeling on consumer choice, research to date has shown mixed results. One large study found that menu labels may help some consumers make lower-calorie selections (2,3), while another small study found no difference in average calories purchased (4).

Menu-labeling laws have the potential to affect not only consumer behavior, but also to prompt industry changes. The prospect of negative consumer reaction to high-calorie menu offerings and increasing consumer demand for lower-calorie menu items may motivate the restaurant industry to reduce portion sizes, alter preparation, and add healthier items to their menus. Restaurants may be particularly sensitive to the nutritional quality of children’s menu items. Public concern regarding the adverse health effects of childhood obesity has already prompted increased scrutiny of children’s meals (5–7) and children’s vulnerability to food marketing (8). In addition, a recent study examined whether calories in adult entrées at fast-food and sit-down chain restaurants in a county with nutrition labeling declined after labeling was implemented (9). The study found that, on average, calories for fast-food chain restaurants decreased by 19 kilocalories (kcal) (standard deviation [SD], 91) over the observation period. However, no trend study has included menus before 2008, the year nutrition-labeling regulations were implemented in New York City, or compared labeled menus with menus from chain restaurants operating in jurisdictions without nutrition-labeling laws.

This exploratory study used 7 years of data, from 2005 through 2011, from a small sample of fast-food chain restaurant companies located in jurisdictions with and without menu-labeling requirements. Our objective was to determine whether the nutritional quality of menus changed over this 7-year time period and if changes differed depending on whether companies were in jurisdictions requiring menu labeling.

## Methods

This case-control trends study examined 7 years of nutritional data, from 2005 through 2011, from 5 chain restaurant companies operating in areas requiring nutrition labeling on menus (cases) and 4 chain restaurant companies operating in areas not yet required to provide nutrition labeling (controls). This study did not include human subjects so was declared exempt by the Drexel University institutional review board.

### Fast-food chain restaurants

Chain restaurants were eligible for inclusion in the study if they were ranked among the top 50 quick-service restaurants in 2010 (10), served entrées (were not coffee shops), and met criteria regarding location and data availability. Restaurant selection aimed to include large restaurant chains, given their larger contribution to Americans’ diets as compared with smaller chains. By using the *Top-50 QSR* (quick service restaurant) ranking (10), restaurants were considered for inclusion beginning with 1 and moving to 50. Location criteria were that the restaurant chain company had 20 or more locations nationally, that case restaurant chains had outlets in areas where menu-labeling regulations had been implemented before 2011, and that control restaurant chains did not have outlets in jurisdictions requiring menu labeling. Data criteria were that the restaurant chain’s website posted nutrition information for 6 or more years of the study period, that nutrition values were listed for 90% or more of menu items, and that values for calories, saturated fat, and sodium were provided (cholesterol and fiber were also provided by the eligible restaurants, so we were able to include them in the analysis). Few chain restaurants consistently posted complete nutrition data during the study period. Only 9 restaurant chains met study criteria and were included (Table 1). For purposes of this article, the 9 restaurant chains included in our study were de-identified and assigned a letter, A through I.

**Table 1:** Characteristics of Fast-Food Chain Restaurants Included in Analysis, 2005–2011

Chain Restaurant^a^	Type of Food	Available Menu Years	Has Children's Menu	Average Number Outlets	Average Revenue^b^ (million $)
**Case chain restaurants**	** NA**	** NA**	** NA**	**3,963**	**$4,074**
Restaurant A	Burger	2005–2011	Yes	3,649	$3,010.00
Restaurant B	Chicken	2006–2011	Yes	5,055	$4,700.00
Restaurant C	Fish	2005–2011	No	964	$700.00
Restaurant D	Burger	2005–2011	Yes	3,572	$3,619.90
Restaurant E	Burger	2005–2011	Yes	6,576	$8,340.00
**Control chain restaurants^c^**	** NA**	**NA**	**NA**	**829**	**$1,081.00**
Restaurant F	Chicken	2005–2011	No	484	$712.80
Restaurant G	Burger	2005–2011	Yes	424	$689.10
Restaurant H	Burger	2005–2008, 2010–2011	Yes	1,692	$1,695.00
Restaurant I	Burger	2006–2011	Yes	717	$1,225.70

a For purposes of this article, the 9 chain restaurant companies included in our study were de-identified and assigned a letter, A through I.

b *QSR* (10).

c Chain restaurants selected as controls were in the following states: Alabama, Arizona, Arkansas, Colorado, Delaware, District of Columbia, Florida, Georgia, Illinois, Indiana, Iowa, Kansas, Kentucky, Louisiana, Maryland, Michigan, Minnesota, Mississippi, Missouri, Montana, North Carolina, North Dakota, Nebraska, New Mexico, Ohio, Oklahoma, Pennsylvania, South Carolina, South Dakota, Tennessee, Texas, Virginia, Utah, West Virginia, Wisconsin, and Wyoming.

### Nutrition values for menu items

Historic data came from an archive of Internet data collected from publicly accessible Web pages beginning in 1996 (11). The archive shows chain restaurant company websites exactly as they appeared in previous years. Dates were printed on each Internet menu; thus, we were able to verify the year the nutritional information represented. When possible, menus were collected from June of each calendar year.

We analyzed sections of the menu with the most food items: à la carte adult entrées, adult side dishes, and à la carte children’s entrées. Adult entrées and sides (including breakfast items) were identified on the basis of their respective menu section headers, and menu sections that were not consistently included in restaurant nutrition data (desserts, beverages, and condiments) were excluded. To reduce the subjective nature of the classification, we retained items listed on the menu as entrées even if they represented less than what would typically be purchased as an entrée (eg, 1 chicken wing). Children’s menu items were identified using the menu section header when available or if the item name contained the words “child” or “kid.” Combination items including an entréeand side were excluded, as were items that specified “family” in the item name (eg, popcorn chicken – family size). Nutrient analysis was limited to calories, cholesterol, fiber, saturated fat, and sodium. Although the majority of existing regulations solely require the posting of calorie information, our analysis included additional nutrients in order to broadly characterize the nutritional quality of fast-food menus and to assess key nutrients of concern in the American diet.

### Summary nutrition values

Restaurants varied in the number of menu items listed annually. To avoid skewing results toward restaurants with numerous items, we calculated the annual mean for each of the 5 nutrition values by restaurant and used that mean to generate annual means for the case and control restaurants. Three menu years were unavailable (Restaurant H, 2009; Restaurant B, 2005; Restaurant I, 2005) and thus were interpolated linearly from available data for each of the restaurants.

### Healthier adult menu items

To determine if restaurant chains altered the proportion of “healthier” menu items, we designated offerings as healthier by using criteria based on Dietary Reference Values (DRV) for a 2000-calorie diet (12), the US Food and Drug Administration standards for packaged food labels (13), and the Dietary Guidelines for Americans (12). There are no federal guidelines for appropriate nutrient levels for adult à la carte restaurant menu items; thus, we selected less than or equal to 25% of the DRV as the criterion for healthier à la carte entrées and less than or equal to 10% of the DRV as the criterion for healthier adult side dishes (Table 2). Some recently published studies used one-third of daily values as a benchmark (9,14) but did not distinguish entrées with and without side dishes. We used one-fourth of daily values because we limited fast-food entrées to those without side dishes (à la carte, not full meals) and because research has shown that most fast-food customers order 2 food items (3). A summary indicator flagged adult menu items that met 4 of 5 nutritional criteria. We originally restricted this indicator to 5 of 5 criteria. However, no menu items satisfied all 5 nutrient criteria, so we relaxed the definition to 4 of 5. Subsequently, we calculated the annual proportion of healthier items for à la carte entrées and sides for each of the 9 restaurants and then aggregated the information for case restaurants and control restaurants.

**Table 2:** Dietary Reference Values and Healthier^a^ Limits for Fast-Food Menu Items

Nutrition value	Calories (kcal)	Cholesterol (mg)	Fiber (g)	Saturated fat (g)	Sodium^b^ (mg)
**Adult dietary reference value** c	2,000	300	25	20	2,300
**Healthier limits for adult fast-food menu items^d^**					
Entrée, à la carte only, 25% DRV	500	75	6	5	575
Side dish, 10% DRV	200	30	3	2	230
**Children's dietary reference value for sedentary/moderately active 8 year old^c^**	1,400	210	18	14	1,610
**Healthier limits for children’s fast-food menu items^e^**					
Entrée, à la carte only, 25% DRV	350	50	4	4	400

Abbreviations: DRV, daily reference values.

a Healthier is defined as foods with DRV at or below 25% for à la carte entrées and at or below 10% for adult side dishes.

b This threshold (2,300 mg) is the tolerable upper limit for persons aged under 40 years and not African-American; the upper limit for most adults is 1,500 mg (12,)

c *Dietary Guidelines for Americans, 2010* (12).

d This study devised a criteria for healthier fast-food menu items because US Department of Agriculture (USDA) does not provide guidelines for adult meals.

e This study devised a criteria for healthier fast-food menu items because USDA only provides guidelines for calories, fat, sodium for children's meals but not each component of those meals.

### Children’s menus

During the study period, 7 of the 9 restaurants listed children’s menus (Table 1). The number of menu items by year and restaurant was small, precluding examination at the restaurant level. Thus, we analyzed children’s menu items by pooling all children's items for the case restaurants and pooling all children’s items for the control restaurants. We based the criteria for children’s menu items on the DRVs for a 1,400-calorie diet, which represents typical calorie needs for sedentary to moderately active children aged 8 years, depending on sex and body size (13); the same criteria have been used by others (6). The US Department of Agriculture, through its school meal programs, has guidelines for calories, fat, and sodium content for children’s meals. However, it does not have standards for each component of a menu. Thus, we applied the following criteria: children’s à la carte entrées were categorized as healthier, as we defined the term, if they were 25% or less of the DRV. Because few children’s items met the “healthier” criteria for fiber or sodium, we used 2 summary indicators, 1 indicator for 3 or more of the 5 nutritional criteria for children’s items and another indicator for 4 or more.

### Statistical analyses

Descriptive analyses were performed on annual means for all nutritional values at the case and control restaurants, annual proportions of healthier menu items, and the case and control differences between first and last year of observed data. Analyses examined each nutrient for items displayed on 9 restaurant menus over 7 years. We estimated case versus control restaurant differences in trends over time for mean nutritional values and proportion of healthier menu items by using the MIXED procedure in SAS version 9.2 (SAS Institute Inc., Cary, North Carolina). Extreme outliers were controlled for by restricting analyses to sections of the menu with the most food items and excluding entrées with sides and entrées that were combination meals. The data were approximately normally distributed, and means and medians approximated each other; therefore, means were reported. Regression models included all years of data, the number of restaurant entrées or sides dishes, and a random intercept for restaurant. The interaction between case status and an indicator variable for pre–post labeling regulation (before vs after 2008) tested whether the outcome variables differed by case status. This procedure was repeated for adult entrées, adult sides, and children’s entrées.

## Results

A total of 4,055 menu items met the eligibility criteria from menus of 9 total case and control restaurants during the period from 2005 through 2011. After removing items that were not entrées or sides (n = 168), the total analytic sample was 3,887 items: 2,529 adult à la carte entrées, 1,186 adult sides, and 172 children’s à la carte entrées.

### Average nutrients in adult menu items

Throughout the study period, across all restaurants, the nutritional profile of fast-food menu items showed high levels of sodium and saturated fat coupled with low levels of fiber (Table 3). When values for an average à la carte entrée and a side dish were combined, they contained 70% and 60% of the daily limit of sodium and saturated fat, respectively. Adult à la carte entrées averaged 450 kcal, 85 mg cholesterol, 3 g fiber, 8 g saturated fat, and 1,100 mg sodium (approximately 2,400 mg sodium per 1,000 calories). Adult sides averaged 300 kcal and 640 mg sodium (or approximately 2,000 mg sodium per 1,000 calories) (Figure). When modeling differences in nutrient averages, regression models found no statistically significant changes over time in nutrient averages and no statistically significant differences between the nutritional averages of case and control restaurants (*P* ≥ .50). Case-by-case analyses highlighted the heterogeneity in restaurant trends (Table 3): 3 of 5 labeled restaurants improved their offerings, while 2 of 5 showed no improvement and even launched new options, such as bacon cheeseburgers, that increased average calories by almost 20% and cholesterol by almost 140%.

**Table 3:** Distribution of Nutrition Values for Adult à la Carte Entrées, Adult Side Dishes, and Children's à la Carte Entrées, by Fast-Food Chain Restaurant, 2005–2011

Menu Section and Restaurant Case Control Status^a^	2005						2011							
**Restaurant**	**n**	**Calories^b^ (kcal)**	**Cholesterol^b^ (mg)**	**Fiber^b^ (g)**	**Saturated Fat^b^ (g)**	**Sodium^b^ (mg)**	**n**	**Calories^b^ (kcal)**	**Cholesterol^b^ (mg)**		**Fiber^b^ (g)**		**Saturated Fat^b^ (g)**	**Sodium^b^ (mg)**
**All chain restaurants**	**320**	**446 (191)**	**82 (61)**	**2.8 (3.5)**	**7.5 (4.6)**	**1,098 (541)**	**380**	**452 (192)**	**92 (72)**		**2.1 (1.5)**		**8.4 (5.6)**	**1,087 (446)**
**Adult à la carte entrees**														
**Case chain restaurants**	**153**	**419 (192)**	**70 (48)**	**3.4 (5.1)**	**6.5 (3.5)**	**1,061 (580)**	**216**	**422 (186)**	**77 (55)**		**2.1 (1.5)**		**6.6 (4.3)**	**1,008 (414)**
Restaurant A	32	525 (164)	65 (38)	1.3 (0.7)	7.0 (3.4)	713 (236)	42	491 (175)	61 (41)		1.4 (0.7)		7.0 (3.4)	738 (254)
Restaurant B	41	415 (211)	98 (78)	6.5 (9.4)	8.6 (3.0)	1,390 (479)	49	289 (162)	64 (25)		2.9 (1.5)		6.6 (3.0)	1,391 (489)
Restaurant C	12	272 (160)	82 (43)	1.8 (1.9)	5.2 (3.2)	1,236 (713)	16	283 (132)	67 (38)		1.4 (1.6)		4.3 (5.4)	833 (467)
Restaurant D	48	503 (298)	46 (33)	1.8 (1.8)	3.9 (2.6)	763 (453)	71	597 (253)	48 (28)		1.6 (1.5)		3.7 (1.9)	773 (370)
Restaurant E	20	379 (127)	61 (49)	5.6 (11.5)	7.9 (5.1)	1,203 (1,021)	38	451 (207)	147 (145)		3.3 (2.0)		11.2 (7.9)	1,303 (491)
**Control chain restaurants**	**167**	**474 (190)**	**94 (74)**	**2.2 (2.0)**	**8.5 (5.7)**	**1,135 (503)**	**164**	**481 (199)**	**108 (89)**		**2.1 (1.6)**		**10.3 (6.8)**	**1,166 (477)**
Restaurant F	20	353 (105)	93 (52)	2.3 (2.0)	10.1 (5.0)	1,235 (440)	16	347 (134)	117 (66)		1.3 (1.2)		13.2 (7.4)	1,101 (430)
Restaurant G	64	516 (173)	110 (93)	1.2 (1.4)	10.6 (9.0)	1,198 (539)	51	552 (197)	104 (110)		2.1 (1.2)		9.3 (7.1)	1,322 (455)
Restaurant H	45	513 (269)	121 (126)	2.5 (1.9)	7.9 (5.1)	1,207 (599)	51	511 (226)	126 (118)		2.4 (1.6)		10.0 (6.5)	1,168 (569)
Restaurant I	38	514 (212)	53 (25)	2.8 (2.7)	5.5 (3.6)	898 (432)	46	515 (240)	83 (60)		2.5 (2.3)		8.7 (6.4)	1,073 (455)
**Adult side dishes**														
**All chain restaurants**	**149**	**295 (140)**	**12 (17)**	**3.1 (2.3)**	**4.1 (3.2)**	**636 (403)**	**184**	**275 (145)**	**11 (15)**		**2.9 (2.0)**		**3.7 (2.9)**	**640 (402)**
**Case chain restaurants**	**75**	**288 (140)**	**11 (16)**	**3.5 (2.2)**	**3.4 (2.6)**	**626 (395)**	**99**	**264 (143)**	**10 (14)**		**3.1 (1.8)**		**3.3 (2.7)**	**568 (385)**
Restaurant A	14	462 (204)	16 (29)	3.6 (1.7)	6.0 (5.1)	960 (612)	23	389 (179)	14 (18)		3.2 (1.7)		4.4 (3.2)	953 (517)
Restaurant B	15	136 (68)	3 (5)	2.1 (2.2)	1.3 (1.0)	394 (289)	18	129 (84)	4 (8)		1.9 (1.9)		1.3 (1.6)	343 (283)
Restaurant C	10	185 (90)	9 (12)	2.1 (1.5)	2.2 (1.2)	395 (219)	15	164 (81)	6 (10)		2.0 (1.1)		2.5 (2.2)	359 (204)
Restaurant D	22	351 (159)	14 (16)	4.4 (2.7)	5.0 (3.6)	987 (487)	32	373 (231)		13 (14)	3.2 (1.8)		5.8 (4.7)	777 (521)
Restaurant E	14	307 (182)	12 (18)	5.2 (2.6)	2.6 (2.1)	392 (365)	11	263 (141)		11 (20)	5.1 (2.7)		2.3 (1.7)	409 (400)
**Control chain restaurants**	**74**	**302 (139)**	**13 (18)**	**2.8 (2.5)**	**4.9 (3.8)**	**647 (412)**	**85**	**287 (146)**		**12 (16)**		**2.7 (2.2)**	**4.2 (3.1)**	**713 (419)**
Restaurant F	14	195 (96)	16 (30)	2.0 (1.6)	2.4 (2.2)	501 (196)	13	200 (98)		18 (31)		2.0 (1.6)	2.6 (2.1)	538 (145)
Restaurant G	36	278 (161)	24 (24)	3.8 (4.5)	5.4 (5.4)	1,073 (571)	44	253 (154)		25 (26)		2.7 (2.4)	4.1 (3.9)	988 (586)
Restaurant H	16	331 (147)	1 (3)	2.6 (2.1)	3.9 (1.8)	525 (320)	18	315 (136)		2 (5)		2.7 (1.6)	3.6 (1.6)	683 (371)
Restaurant I	8	404 (151)	9 (14)	2.6 (1.6)	7.6 (5.9)	490 (560)	10	380 (198)		2 (4)		3.6 (3.0)	6.6 (4.9)	643 (574)
**Children's à la carte entrees**														
**All chain restaurants**	**22**	**265 (20)**	**37 (11)**	**1.5 (0.9)**	**5.7 (0.8)**	**796 (91)**	**23**	**313 (62)**		**37 (5)**		**1.4 (0.8)**	**4.6 (0.9)**	**754 (159)**
**Case chain restaurants**	**12**	**261 (8)**	**31 (10)**	**1.1 (0.5)**	**3.8 (0.3)**	**750 (132)**	**12**	**255 (25)**		**31 (1)**		**1.3 (0.4)**	**3.5 (1.1)**	**638 (95)**
**Control chain restaurants**	**10**	**491 (152)**	**44 (12)**	**2.0 (1.3)**	**7.6 (1.3)**	**842 (49)**	**11**	**372 (99)**		**43 (10)**		**1.6 (1.3)**	**5.8 (0.8)**	**871 (222)**

a For the purposes of this article, the 9 chain restaurant companies included in our study were de-identified and assigned a letter, A through I.

b All values are mean (standard deviation) unless otherwise noted.

**Figure:**
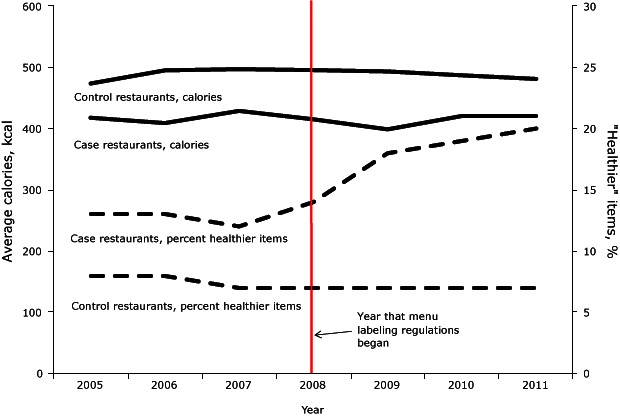
Average calories for à la carte entrées and percentage of healthier items on fast-food chain restaurant menus, by case and control status and year, 2005–2011. Healthier à la carte entrées are defined as DRV ≤ 25%. Calorie-labeling regulations were first introduced in 2008. **Table:** 

Average Calories (kcal)	2005	2006	2007	2008	2009	2010	2011
Case chain restaurant	419	410	429	415	400	422	422
Control chain restaurant	474	495	497	496	494	487	481
Healthier items (%)							
Case chain restaurant	13	13	12	14	18	19	20
Control chain restaurant	8	8	7	7	7	7	7

### Adult healthier menu items

During the study period, the overall proportion of healthier adult entrées that met 4 or more of the nutritional guidelines remained less than 25%, with fiber and sodium least likely to meet healthier standards (Table 4). Control restaurants had a lower proportion of healthier items than cases, remaining at approximately 7% over the study period (Figure). In contrast, case restaurants increased the proportion of healthier entrées after labeling regulations: from 13% during years 2005 through 2008, up to 20% by 2011 (regression trend analysis found a mean difference of 5% pre–post 2008 in cases relative to controls: interaction, β = 5.1%; 95% confidence interval [CI], 0.9%–9.3%, *P* = .02). These improvements were largely attributed to Restaurant A (reduced saturated fat), Restaurant B (offered grilled alternatives to deep-fried entrées, and reduced calories and sodium) and Restaurant D (reduced calories). The prevalence of healthier side dishes was higher among case restaurants than controls (23% vs 15%, respectively) and did not change over time. Similar to entrées, sodium levels in sides were least likely to meet the healthier standard.

**Table 4:** Percentage of Menu Items Meeting Healthier^a^ Nutritional Values for Adult à la Carte Entrees, Adult Side Dishes, and Children's à la Carte Entrees, by Fast-Food Chain Restaurant, Years 2005 and 2011

Case Control Status^b^	2005						2011					
	Met 4 of 5 criteria (%)	Calories (%)	Cholesterol (%)	Fiber (%)	Saturated Fat (%)	Sodium (%)	Met 4 of 5 criteria (%)	Calories (%)	Chol-esterol (%)	Fiber (%)	Saturated fat (%)	Sodium (%)
**Adult à la carte entrees**	11	66	64	5	41	16	13	67	60	2	41	17
**Case chain restaurants**	13	70	73	8	50	16	20	70	67	3	53	21
Restaurant A	3	50	59	16	16	3	7	57	76	2	33	7
Restaurant B	12	68	68	2	59	15	27	88	63	0	78	31
Restaurant C	25	92	75	0	83	33	44	94	94	0	88	44
Restaurant D	6	58	77	10	44	10	8	42	46	7	27	8
Restaurant E	20	80	85	10	50	20	13	68	55	5	39	13
**Control chain restaurants**	8	62	55	3	32	15	7	64	53	1	28	13
Restaurant F	10	90	60	0	42	35	13	88	75	0	42	38
Restaurant G	6	53	47	8	22	3	4	49	31	0	12	4
Restaurant H	7	56	53	0	29	16	4	65	55	0	33	4
Restaurant I	8	50	61	5	37	8	7	57	50	2	26	7
**Adult sides**	18	35	87	55	36	22	19	40	89	47	38	18
**Case chain restaurants**	22	40	89	63	44	20	23	43	89	49	43	23
Restaurant A	0	0	79	64	14	0	4	13	83	61	30	9
Restaurant B	40	87	100	33	87	33	39	78	94	22	78	39
Restaurant C	30	70	100	50	50	20	33	67	100	40	47	27
Restaurant D	14	14	86	86	32	9	13	31	88	50	25	13
Restaurant E	29	29	79	79	36	36	27	27	82	73	36	27
**Control chain restaurants**	14	31	85	47	28	25	15	37	88	44	33	13
Restaurant F	21	57	86	29	50	7	15	54	85	31	46	0
Restaurant G	14	36	67	53	31	22	18	45	68	36	45	23
Restaurant H	6	19	100	56	19	19	6	28	100	50	22	11
Restaurant I	13	13	88	50	13	50	20	20	100	60	20	20
**Children's à la carte entrees^c^**	4	71	85	0	34	4	8	69	86	0	38	8
**Case chain restaurants**	8	92	100	0	58	8	17	92	100	0	67	17
**Control chain restaurants**	0	50	70	0	10	0	0	45	73	0	9	0

a Healthier is defined as foods with daily recommended values at or below 25% for à la carte entrees and at or below 10% for adult side dishes.

b For purposes of this article, the 9 chain restaurant included in our study were de-identified and assigned a letter, A through I.

c When the definition of healthier children’s items was relaxed so that the criteria were 3 or more of the 5 nutrition criteria (rather than 4 or more as shown in the table), prevalence of healthier children’s à la carte entrees among all restaurants was 39% (2005), 42% (2011); among case restaurants, 72% (2005), 79% (2011); and among control restaurants, 6% (2005), 6% in (2011).

### Children's menu items

Average nutritional values for children’s à la carte entrées at case restaurants were 260 kcal and 690 mg sodium and at control restaurants, 430 kcal and 860 mg sodium. Pooled across all restaurants, children’s à la carte entrées averaged 37 mg cholesterol, 2 g fiber, and 5 g saturated fat. By using 4 or more of the 5 nutritional criteria to define healthier options, no control restaurants had qualifying items, and only 1 case restaurant had qualifying items (Restaurant E). When healthier options were defined as 3 or more out of the 5 nutritional criteria, the proportion of healthier children’s entrées at case restaurants was much higher than at control restaurants. However, no difference was observed in the trend over time: prevalence for meeting 3 of 5 criteria was around 60% for case restaurants and 10% for control restaurants (difference in prevalence, n = 22, *P* = .02; test for interaction in regression trend analysis, *P* = .60). Similar to adult menu items, fiber and sodium in children’s menu items were least likely to meet healthier criteria.

## Discussion

We found that after the implementation of menu labeling there was a statistically significant increase in the percentage of healthier adult entrées at restaurants in jurisdictions with menu-labeling laws compared with restaurants that were not in jurisdictions subject to labeling. Little improvement, however, was seen among children’s entrées during this period, and no significant changes in average nutritional values were seen among adult entrées and sides. Our results suggest menu labeling may provide fast-food restaurants with motivation to introduce healthier menu options; however, greater pressure may be necessary to generate overall average nutritional improvements.

Two recently published articles, by Bruemmer et al (9) and Saelens et al (15), used data from October 2008 through July 2010 to evaluate short-term changes in the nutritional quality of menu items after labeling was enacted in Seattle, King County, Washington. As we found in our study, few substantive improvements were made in fast-food restaurant menus during the study period (9,15). Bruemmer et al’s, case-only study design included a reformulation analysis (items that remained on the menu during the study period) that found a statistically significant decrease in fast-food calories, but with small differences; when all fast-food items were analyzed, they found no improvements. In contrast to our study, neither the Bruemmer or Saelens study found improvements in the percentage of fast-food menu options that were healthier (9,15). Differences between those studies and ours included study design, selection of fast-food chains (their studies included more types of fast-food menus: burger, pizza, sandwich, Tex-Mex), sample size, data years, and criteria for healthier items (9,15). We studied a longer time period (2005–2011), including 3 years before New York City implemented its regulations, and we used as our control group chains that had never had to comply with local menu-labeling regulations. To define “healthier” items, other studies used a calorie cut point set to one-third of daily recommended limits (9) or criteria that were specific to a restaurant audit instrument (15), whereas we used a composite index derived from recommendations for calories, cholesterol, fiber, saturated fat, and sodium and set the criteria at one-quarter of daily limits.

To date, menu-labeling regulations have focused primarily on calories. Nutrient profile information reported in this study suggests that policies to address other key nutrients in fast food may have value. At all time periods and at both case and control restaurants, sodium levels remained consistently high, far exceeding healthy limits, and dietary fiber was very low. Diets high in sodium can increase risk of hypertension and cardiovascular disease (16). Dietary fiber has been found to be protective of numerous health conditions (17) and can aid weight reduction by reducing the energy density of foods, while maintaining portion size (18). Fast-food restaurants need to increase fiber in their offerings and be more creative in reformulating their foods to reduce sodium (19).

Study limitations included our reliance on information that fast-food chain restaurant companies posted on their Web pages. However, Web data are quite good representations of actual values: a validation study found only small differences between nutrition information posted on fast-food chain restaurant websites and laboratory analyses (average difference was <10 kcal) (20). A key strength of our study was that we controlled for secular trends by comparing case restaurants with control restaurants (located in jurisdictions outside areas with menu labeling), and we were able to assess the nutritional value of the foods over a long time period (7 years). The restaurant chain companies included in this study have a large presence in the US market, representing more than 23,000 US outlets and reporting $24.7 billion in sales for 2011. However, only 9 chains were eligible because they posted sufficient breadth of historical nutritional information. Therefore, trends observed in this study may not be generalizable to other large chains or different menu types. High heterogeneity within and between restaurants challenged our ability to statistically and substantively assess change. Several steps were taken to reduce high heterogeneity: using menu sections with a large number of menu items that were likely to be similar (example: à la carte entrées), and regression that used restaurant-specific intercepts and controlled for number of items on the menu. Nevertheless, entrée portion sizes varied within and between restaurants, from a single drumstick or fish filet to a large bacon cheeseburger.

In summary, our results in combination with other recently published studies suggest that menu-labeling policies may have already motivated some changes in some chain restaurant menus; however, menus have not markedly improved overall. This study provides a preliminary look at changes in fast-food menus when restaurants first began implementing labeling, but more studies are needed over longer time periods and with larger sample sizes. When menu labeling is required nationally (21), our understanding of industry response to menu labeling will likely become clearer and may be different once all large chains are required to label and customers become accustomed to having nutrition information readily available in chain restaurants. Additional public policies and media advocacy campaigns may be needed to spur broader changes in restaurant offerings so that healthier restaurant choices become the default choice for consumers (22). In addition, policy makers could consider minimum nutritional standards for meals targeted to children, as have been implemented in a few local jurisdictions (23,24). Simultaneous strategies should be considered to encourage chain restaurant companies to significantly improve the nutritional quality of the foods they sell, with portion-size reduction a key focus.
